# Activation of focal adhesion kinase enhances the adhesion of *Fusarium solani* to human corneal epithelial cells via the tyrosine-specific protein kinase signaling pathway

**Published:** 2011-03-05

**Authors:** Xiaojing Pan, Ye Wang, Qingjun Zhou, Peng Chen, Yuanyuan Xu, Hao Chen, Lixin Xie

**Affiliations:** 1 State Key Laboratory Cultivation Base, Shandong Provincial Key Laboratory of Ophthalmology, Shandong Eye Institute, Qingdao, China; 2 Department of Ophthalmology, Affiliated Hospital of Qingdao University, Qingdao, China

## Abstract

**Purpose:**

To determine the role of the integrin-FAK signaling pathway triggered by the adherence of *F. solani* to human corneal epithelial cells (HCECs).

**Methods:**

After pretreatment with/without genistein, HCECs were incubated with *F. solani* spores at different times (0–24 h). Cell adhesion assays were performed by optical microscopy. Changes of the ultrastructure were observed using scanning electron microscopy (SEM) and transmission electron microscopy (TEM). The expression of F-actin and Paxillin (PAX) were detected by immunofluorescence and western blotting to detect the expression of these key proteins with/without genistein treatment.

**Results:**

Cell adhesion assays showed that the number of adhered spores began to rise at 6 h after incubation and peaked at 8 h. SEM and TEM showed that the HCECs exhibited a marked morphological alteration induced by the attachment and entry of the spores. The expression of PAX increased, while the expression of F-actin decreased by stimulation with *F. solani*. The interaction of *F. solani* with HCECs causes actin rearrangement in HCECs. Genistein strongly inhibited FAK phosphorylation and the activation of the downstream protein (PAX). *F. solani-*induced enhancement of cell adhesion ability was inhibited along with the inhibition of FAK phosphorylation.

**Conclusions:**

Our results suggest that the integrin-FAK signaling pathway is involved in the control of *F. solani* adhesion to HCECs and that the activation of focal adhesion kinase enhances the adhesion of human corneal epithelial cells to *F. solani* via the tyrosine-specific protein kinase signaling pathway.

## Introduction

Fungal keratitis is a common blinding disease, which over the past decades, has had an increased incidence in many agricultural countries [1]. The dominant filamentous fungal pathogens are Fusarium species, with *Fusarium solani* (*F. solani*) being the most frequent isolate among the Fusarium species of keratomycosis in north China [1,2]. Poor knowledge of the pathogenesis of this disease makes effective treatment difficult [2]. Previous studies suggest that the interaction between host cells and fungus may play a critical role in the pathogenesis of fungal diseases [3-5]. Our previous work [6] demonstrated the roles of adherence and matrix metalloproteinases (MMPs) in growth patterns of major fungal pathogens (including *F. solani*) in the cornea. However, the precise molecular mechanism in keratomycosis remains unknown. Furthermore, several phosphate-containing proteins have been shown in many cancer cells [7] and gastric epithelial cells [8], the stimulation of tyrosine phosphorylation by several substrates correlates with increased adhesion, motility, invasion and alteration in the cytoskeleton, and overexpression and phosphorylation of focal adhesion kinase (FAK) in epithelial cells promotes adherence to Candida yeast cells [9]; however, the role of FAK and tyrosine phosphorylation in the regulation of the interaction of human corneal epithelial cells (HCECs) with *F. solani* has been poorly elucidated. Therefore, the study of signal transduction pathways in HCECs stimulated by *F. solani* is especially important in view of their putative implications in the regulation of interaction.

Adherence to host cells, such as endothelial and epithelial cells, is the first step in colonization by fungus and the subsequent establishment of infection [10-12]. Similarly, the adherence of fungus to epithelial cells and to extracellular matrix (ECM) components is considered a crucial event in pathophysiology [9,13-15]. In other issues, multiple adhesions, such as mannoproteins, lectin-like receptors, carbohydrates and integrin-like molecules, can mediate fungus-host cell adhesion [13,16]. Integrins are a large family of highly conserved heterodimers composed of noncovalently linked α and β subunits that mediate cell-matrix and cell-cell interactions in embryogenesis, hemostasis, wound healing, tumor and microorganism invasion, immune response, and inflammation [8]. These receptors mediate the tight adhesion of cells to the ECM at sites referred to as focal adhesions. Within focal adhesions, the cytoplasmic domains of the integrin heterodimers provide a site to which cytoskeletal proteins are tethered.

The FAK family consists of two evolutionarily conserved protein tyro-kinase (PTKs) localized in the focal adhesions, namely, p125 focal adhesion kinase (p125FAK) and proline-rich tyrosine kinase 2 (Pyk-2) [17,18]. Several studies have shown that FAK functions as part of a cytoskeletal-associated network of signaling proteins, including paxillin (PAX), Src (a proto-oncogenic tyrosine kinase)-homology collagen (Shc), and growth factor receptor-bound protein 2 (Grb-2), which act in combination to transduce integrin-generated signals to mitogen-activated protein kinase (MAPK) cascades [4,5,18].Tyrosine phosphorylation of the FAK family is regulated by different stimuli, which include adhesive events, in that several components of the ECM, such as fibronectin (FN), vitronectin (VN), laminin [19], and collagen IV, or clustering of β1, β3, and β5 integrins, trigger p125FAK tyrosine phosphorylation.

Recently available information suggests that integrin-FAK is one of the best characterized intracellular signaling pathways, which play a critical role in the control of cell adherence, migration, and internalization when activated by a series of stimuli [20]. It seems likely that the activation of the integrin-FAK signaling pathway may be involved in the interaction between fungus and cell surface receptors responsible for transmitting downstream signals. Fungus might associate either directly or indirectly with integrin to modulate FAK and downstream signals leading to cell adherence and migration. The present study sought to determine whether a putative p125FAK that is expressed in HCECs co-culture with *F. solani* and whether cross-linking of the β1 integrin receptors or adhesion to HCECs can regulate tyrosine phosphorylation. Furthermore, we investigated the mechanism of activation of FAK and its downstream PAX signaling following adhesion to ECM. Our results suggest that activation of FAK enhances the adhesive and migration capabilities of HCECs through the tyrosine-specific protein kinase signaling pathway.

## Methods

Unless otherwise stated, all chemicals used were of analytical grade or higher. The tyrosine-specific protein kinase signaling pathway inhibitor, genistein, was purchased from Sigma-Aldrich Shanghai Trading Co. Ltd. (Shanghai, China). DMEM/F-12 (1:1) was purchased from Thermo Fisher Scientific (Beijing, China).

### Strains and culture conditions

The strain of *Fusarium solani* (CGMCC 3.1829) was purchased from China General Microbiologic Culture Collection Center, (Beijing, China). The two strains were cultured on potato dextrose agar (PDA; Qingdao Hope Bio-Technology Co. Ltd., China) at 28 °C for 5 days, and spores were harvested into 1 ml sterile saline solution and then diluted with sterile saline to yield 10^8^ U/ml (culturable).

### Cell adhesion assay

Simian Virus 40-immortalized HCECs were used in the present study [21]. They were kindly gifted by Dr. Choun-K_i_ Joo (Catholic University of Korea, Seoul, Korea). The cells were maintained in DMEM/F 12, 5% fetal bovine serum (FBS), 100 IU of penicillin/ml, and 100 mg of streptomycin/ml in a humidified 5% CO_2_ incubator at 37 °C. The HCECs were pretreated with/without genistein (200 μΜ) [22] for 1 h and then incubated with fungi at different times (0 to 24 h). Untreated monolayers (controls) were incubated in DMEM/F12. Adhesion was verified microscopically every hour and the spore’s phases were maintained throughout the adhesion assays. Unattached fungi were removed by extensive washing with PBS. The number of fungi spores was counted and the results were analyzed by the measurement of integral optical density (IOD) with an image analyzer (Vidas 21; Kontron Corp., Eching, Germany). Experiments were repeated at least three times.

### Electron microscopy

The protocol for scanning electron microscopy (SEM) and the protocol for transmission electron microscopy (TEM) are at specific websites. After incubation with fungi spores for different times, the cells were washed three times with PBS. Then, the cells were fixed in 4% buffered glutaraldehyde, washed in a buffered solution of 0.2% sucrose-kakodyl for 4–10 h, and dehydrated in graded alcohol concentrations. For SEM (JEOL JSM-840; JEOL, Tokyo, Japan), the specimens were replaced with isoamyl acetate, air-dried, and sputter-coated with gold before examination under the microscope. For TEM, semi-thin sections (1 µm in thickness) of the specimens were embedded in an epoxy resin for orientation purposes and were subsequently stained with toluidine blue. In addition, ultrathin sections were stained with uranyl acetate-lead citrate and were examined on a JEOL JEM-1200 transmission electron microscope (JEOL). The central and paracentral regions were also observed.

### Flow cytometry

The effect of fungi spores on β1 integrin expression in HCECs was determined by flow cytometry (FCM) analysis. Briefly, cells treated as described above were harvested from the 6-well plates following treatment with trypsin. The cell suspension, at a concentration of 1.0×10^6^ cells/ml, was fixed for 20 min in 40 mg/l paraformaldehyde, blocked for 20 min at room temperature in 1% BSA, washed twice in cold PBS, and stained overnight at 4 °C with a rabbit monoclonal anti-β1 integrin antibody. The bound antibody was visualized with a fluorescein isothiocyanate (FITC)-conjugated secondary antibody at room temperature for 1 h and washed three times with PBS. The labeled cells were determined over 10,000 events by flow cytometry (BD FACSCalibur; Becton Dickinson, San Jose, CA) and analyzed using CellQuest Pro Software (Becton Dickinson).

### Immunofluorescence

The expression of PAX and F-actin were shown by immunofluorescence. The cells were fixed in 40 mg/l paraformaldehyde for 20 min, blocked for 10 min at room temperature in 1% BSA, and then incubated overnight at 4 °C with the appropriately diluted primary antibody. In addition, normal rabbit IgG or mouse IgG was used as a negative control. The bound antibody was visualized with a fluorescent secondary antibody at room temperature for 1 h, following standard protocols. Finally, the cells were covered with mounting media (Ultra Cruz^TM^ Mounting Medium; DAPI; sc-24941; Santa Cruz Biotechnology, Santa Cruz, CA) and examined by fluorescence microscopy (Eclipse TE2000-U; Nikon, Tokyo, Japan). Phalloidin-FITC (ALX-350–268-MC01; Alexis Biochemicals, Lausanne, Switzerland) was also used to observe the changes in the cellular cytoskeleton.

### Western blot analysis

Protein was extracted from the HCECs using RIPA lysis buffer (50 mM Tris PH 7.4, 150 mM NaCl, 1%Triton X-100, 1% sodium deoxycholate, 0.1% SDS, sodium orthovanadate, and sodium fluoride; Galen, Beijing, China) according to the manufacturer’s instructions. Each of the prepared samples, in a final volume of 15 μl (containing a total of 50 μg of protein), were run on a 10% SDS–PAGE and then transferred into a polyvinylidene difluoride (PVDF) membrane (Millipore, Billerica, MA). The blots were blocked in 5% non-fat dry milk dissolved in TBST (20 mM Tris PH 7.5, 0.5 mM NaCl, 0.05%Tween-20) for at least 1 h and incubated with the primary antibody in TBST for 1 h at room temperature. Subsequently, the blots were incubated for 1 h at RT with a horseradish peroxidase-conjugated secondary antibody in TBST. The membranes were then developed with a SuperSignal West Femto Maximum Sensitivity substrate (Pierce Biotechnology, Rockford, IL) and exposed to X-ray film (Kodak, Rochester, NY). Immunoreactive bands were visualized via chemiluminescence and quantified using NIH Image 1.62 software (National Institutes of Health, Bethesda, MD). The primary antibodies were rabbit monoclonal anti-p-FAK antibody (ab4803; Abcam, Cambridge Science Park, Cambridge, UK), rabbit polyclonal anti-FAK antibody (cst-3285; Cell Signaling Technology, Beverly, MA), rabbit monoclonal anti-β1 integrin antibody (ab52971; Abcam), and goat polyclonal anti-p-PAX antibody (sc-14036; Santa Cruz Biotechnology).

### Statistical analyses

The statistical differences of each sample comparing the treated and experimental groups were analyzed using the one-way ANOVA (ANOVA) and Student-Newman-Keul's (SNK) test. All p values less than 0.05 were considered statistically significant.

## Results

### Involvement of FAK with adhesive capabilities of HCECs to *F*. *solani* spores

**Figure 1:**
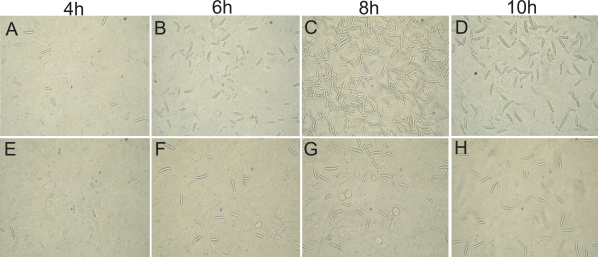
Comparison of the number of *F. solani* spores adhered to HCECs. After pretreatment with/without 200 μM genistein for 1 h, cells were incubated with *F. solani* spores for different times (0 to 24 h). In the *F. solani* and HCEC co-incubated group, the number of adhered spores began to rise at 6 h after incubation, peaked at 8 h, and maintained at over 10 h (**A**-**D**). In the genistein treated group, the adhesiveness was reduced compared with the genistein non-treated HCECs (**E**-**H**).

**Figure 2:**
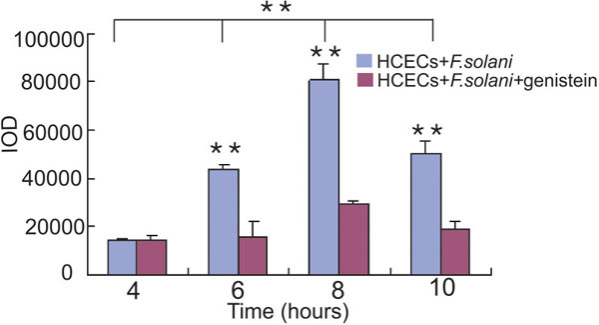
The comparison of optical density (IOD) levels between the *F. solani* and HCEC co-incubated group and the genistein treated group. Spore adhesion assays were performed by measuring the IOD. Statistical significance was tested by one-way ANOVA and Student-Newman-Keul's test. The p values indicated significant differences between the data in the experimental groups (6, 8, and 10 h) and their corresponding treated groups. **p<0.001.

After incubation with HCECs for different time points (0 to 24 h), the spores were respectively observed by light microscopy. In the *F. solani* and HCEC co-incubated group, the number of adhered spores began to rise at 6 h after incubation, peaked at 8 h, and maintained over 10 h compared with the HCECs (Figure 1). Then, the effects on the adhesive response in HCECs to *F*. *solani* spores were investigated along with the inhibition of FAK tyrosine phosphorylation. Inhibition with genistein showed a reduced adhesiveness to spores. When genistein treated HCECs interacted with the spores, a reduced adherence of the spores was observed in comparison to the untreated cells (Figure 1). The result was determined by measurement of the IOD (Figure 2). These data suggest that FAK regulation may play a critical role in the adhesive capabilities of HCECs to *F. solani* spores.

### Ultrastructure

**Figure 3:**
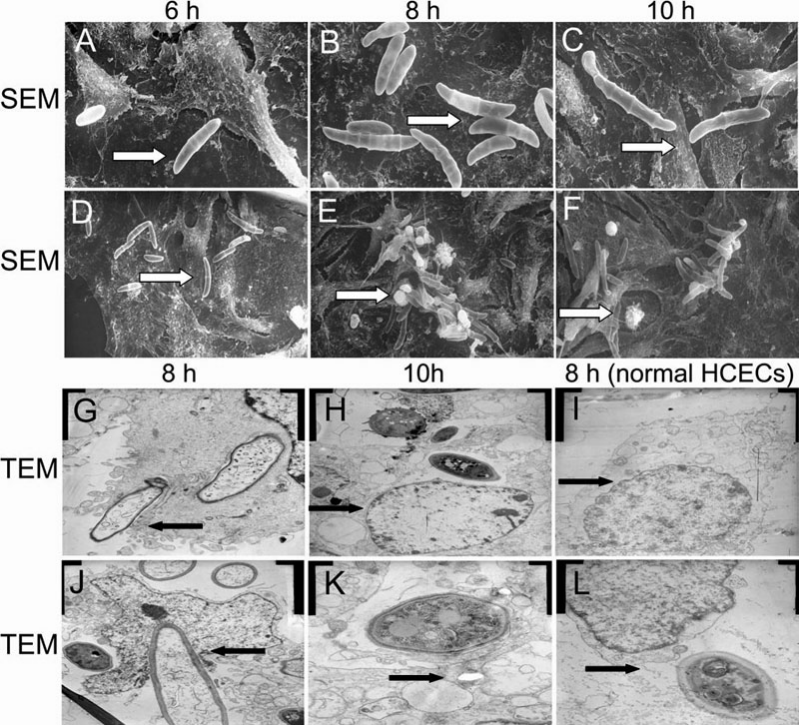
Changes in the ultrastructure after HCECs were coincubated with *F*. *solani* spores. **A**-**D**: Scanning electron micrographs of HCECs coincubated with *F. solani* spores. After coincubation for 6 h, the *F. solani* spores (arrow) began to attach to the surface of the HCECs as seen in panels (**A**) and (**D**). The number of adhered cells increased at 8 h after coincubation (**B**, arrow). Most of the cells presented a great number of spores congregated to the projections of plasma membrane (**A**-**E**, arrow). Damage to the HCECs can be seen in panels **B**, **E**, **C**, and **F**. Ruptured and extensively destroyed membrane with spores adhering to it (**C**, arrow) and characteristics of dead cells (**F**, arrow) at 10 h after coincubation. Original magnification: (**A**, **B**, and **C**) 2,000×; (**D**, **E**, and **F**) 800×. **G**-**L**: Transmission electron micrographs of cells incubated with HCECs and *F. solani* spores. Photograph shows *F. solani* spores attached to the plasma membrane, followed by the subsequent formation of cell projections around it (8 h, **G**, and **J**, arrow). Then, spores are internalized into the cell cytoplasm and cell organelles appeared to be destroyed at 10 h (**H** and **I**). The ultrastructures of most HCECs show confused organelle structure degeneration of the nucleus (**H**, arrow), and vacuolization of the mitochondria (**K**, arrow) at 10 h after incubation. Original magnification: (**G**) 8,000×; (**I** and **J**) 5,000×; (**H**) 3,000×; (**K**) 20,000×; (**L**) 12,000×.

Examination of the SEM images showed that the normal HCECs had uniform epithelial cell morphology with numerous microvilli located on the surface. Adhesion, as determined at different times (6, 8, and 10 h) after cell incubation and observed by the cells with attached spores, is shown in Figure 3A-F. In the *F. solani* and HCEC co-incubated group, the morphology of the HCECs was characterized as being corrugativus and pantomorphic where the microvilli were fewer in number. Such changes were more significant in the 8 h and 10 h groups (Figure 3B,E,C,F). After 10 h adhesion, the cellular areas were even smaller and the microvilli were less numerous than those observed at 8 h. Most of the HCECs retained the characteristic ultrastructures of the ruptured membranes and shrunken and dead cells (Figure 3F). It is interesting to note the obvious clumping of adherent spores attached to the filament-like projections stretching from the plasma membrane (Figure 3E). These clumps were evident after 8 h and contained numerous spores after 10 h of incubation. Ruptured and extensively destroyed membrane with spores adhering to it and the characteristics of the dead cells were also apparent at 10 h after inoculation.

In addition, TEM was used to examine the ultrastructural features of the HCECs at 6, 8, and 10 h after incubation with the *F. solani* spores (Figure 3G-L). The TEM images show that the normal cells arrayed with the monolayer and took on a polygon shape. Their organelles, such as mitochondria and rough endoplasmic reticulum, were abundant. The nuclear membranes were full and slick and the nuclei were large (Figure 3I,L). During the adherence process, the HCECs came into interact with spores instead of fusing with them (Figure 3G,J). Damage to the HCECs can be seen at 6 h. After 8 h incubation, most cells exhibited a marked morphological alteration. The normal organelles were significantly less well resolved and the vacuoles were larger and more abundant in the cytoplasm (Figure 3H,K). Curiously, at 10 h, some of the HCECs started to die. *F. solani* spores could be observed inside cells at 10 h after incubation (Figure 3H,K).

### Expression of β1 integrin on HCECs evaluated by flow cytometry

**Figure 4:**
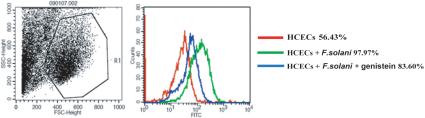
Flow cytometry analysis results for the expression of β1 integrin. The data evaluated the expression of β1 integrin on permeabilized HCECs by flow cytometry. As shown in the figure, the anti-β1 integrin, mAb, positively stained cells both in the group incubated with *F. solani* spores and in the genistein pretreated groups (97.97% and 83.60%, respectively), while 56.43% of the cells were positive in the negative controls. Significant differences were observed between the above two groups. The results were representative of one of three separate experiments.

We further evaluated the expression of β1 integrin on HCECs by flow cytometry. β1 integrin expression on cell surfaces was significantly increased when the cells were treated with *F. solani* spores or genistein. The expression of β1 integrin increased by 97.97% when the cells were incubated with *F. solani* spores for 8 h (Figure 4). However, when the cells were pretreated with genistein, the expression of β1 integrin decreased by 83.60%. However, no significant differences were found in the two groups (p>0.05).

### Expression of PAX and F-actin

**Figure 5:**
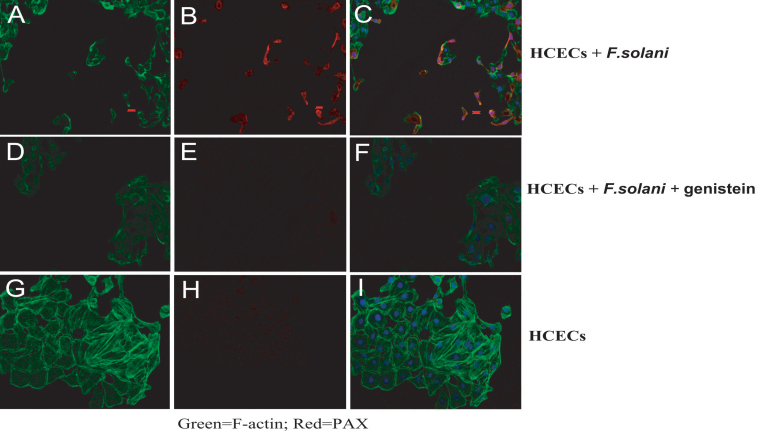
Immunofluorescence and confocal microscopy were used to detect the expression of PAX and F-actin. F-actin was stained with FITC (**A**, **D**, and **G**) and PAX was stained with Texas red (**B**, **E**, and **H**). The areas of co-localization appear yellow in the merged sections (**C**, **F**, and **I**). When the cells were pretreated with genistein, the expression of PAX decreased (**B**, **E**, and **H**). Incubation with *F. solani* spores induced alterations in the F-actin microfilaments of the HCECs (**A**, **D**, and **G**). These results showed that the untreated HCECs exhibited normal morphology, while an actin rearrangement was noted in cells incubated with the *F. solani* spores. A combined treatment with genistein and the spores decreased the polymerization of actin, and the HCECs became more spreading than in the group incubated with *F. solani* spores.

Immunofluorescence and confocal microscopy were used to detect the expression of PAX and F-actin. F-actin was stained with FITC (Figure 5A,D,G) and PAX was stained with Texas red (Figure 5B,E,H). The areas of co-localization appear yellow in the merged sections (Figure 5C,F,I). When the cells were pretreated with genistein, the expression of PAX decreased (Figure 5B,E,H). Incubation with *F. solani* spores induced alterations in the F-actin microfilaments of the HCECs (Figure 5A,D,G). These results show that the untreated HCECs exhibited normal morphology, while an actin rearrangement was noted in cells incubated with the *F. solani* spores. A combined treatment with genistein and the spores decreased the polymerization of actin, and the HCECs became more spreading than in the group incubated with *F. solani* spores. These results indicate that the inhibition of FAK signaling alters cell-spore interaction.

### Western blot analysis of the integrin-FAK signaling pathway in HCECs

**Figure 6:**
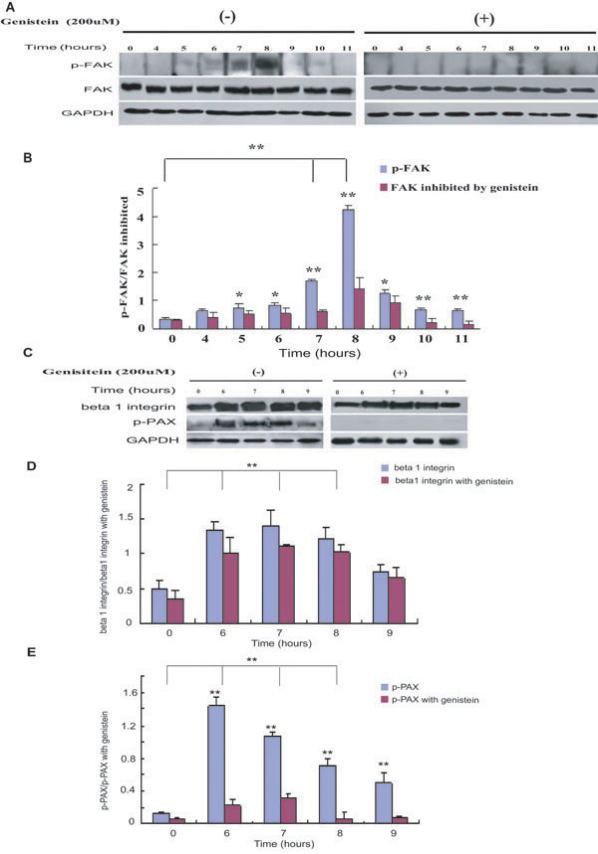
Involvement of FAK phosphorylation and integrin signaling with adhesion of HCECs to *F. solani* spores. After pretreatment with/without genistein, HCECs were exposed to *F. solani* spore suspensions. Western blot assay showed that 5 h after incubation, p-FAK production was significantly increased (**A**). The graph (**B**) compares scanning signal intensity of p-FAK expression by ImageJ software. The expression of p-FAK greatly increased (p<0.001) in all treated groups (7 and 8 h). There were significant differences for the phosphorylation levels of FAK in all treated groups. *p<0.01, **p<0.001. The β1 integrin and p-PAX from cells incubated with *F. solani* spores were also analyzed by western blot (**C**). The graph (**D**) compared scanning signal intensity of β1 integrin expression by ImageJ software and indicated the significant overexpression (p<0.001) of β1 integrin protein in all treated groups (6, 7, and 8 h). The data showed no significant differences (p<0.05) between the genistein treated and non-treated groups. The expression of p-PAX was significantly increased (p<0.001) in all treated groups and when the cells were pretreated with genistein, the expression of p-PAX was significantly lower (p<0.001) than in the no-genistein treated group (**E**). GAPDH was used as a loading control.

The FAK proteins are the key proteins of the FAK signaling pathway, which is phosphorylated and subsequently activated. To further understand the state of activity of the integrin-FAK signal cascade as a key position of the adherence of *F. solani* spores to the HCECs, we compared the expression levels of the p-FAK, p-PAX, and β1 integrin proteins in the genistein pretreatment group and the group incubated with *F. solani* spores. Incubation of HCECs with *F. solani* spores stimulates FAK tyrosine phosphorylation at 7 h after incubation. The expression reached a peak after 8 h incubation and decreased at 9 h. Pretreatment of cells with genistein downmodulated FAK tyrosine phosphorylation induced by the spores’ interaction with the cells (Figure 6A,B). The data also show that the expressions of the p-PAX and β1 integrin proteins both increase at 6 h in the experimental groups and downregulation of p-FAK inhibits p-PAX expression in the HCECs (Figure 6C,D,E).

## Discussion

Keratitis caused by *F. solani* usually occurs following corneal injury. Pioneering work identified that injury predisposes the cornea to infection by permitting this organism to adhere to it. Adherence is not immediate and requires that the organisms remain on the corneal surface for some time [1,6]. Furthermore, sparse information is available regarding the signaling events triggered by the contribution of *F. solani* to the pathogenesis of corneal infection. Here, we provide the first evidence of the presence of a focal adhesion kinase (FAK) protein and its involvement in the control of integrin-mediated *F. solani* spore adhesion.

Tyrosine phosphorylation of cellular proteins is a primary response to integrin stimulation and the role of the PTKs belonging to the FAK family in the control of cellular adhesion and migration is documented [18,19,23]. Our findings suggest that the enhancement of adhesion of *F. solani* to HCECs is dependent on the presence of β1 integrin and tyrosine phosphorylation of FAK, which is obviously blocked by using the PTK inhibitor, genistein, pretreatment [9]. These results are in line with previous evidence in other cells where cell adhesion to ECM or clustering of β3 and β5 integrins triggered p125 FAK tyrosine phosphorylation [24,25].

To examine the role of the integrin-FAK signaling pathway in the adhesion of HCECs, we examined the signaling molecules that were involved in mediating spore-induced effects on cells and investigated whether β1 integrin is physically associated with FAK/PAX or whether the activation of β1 integrin-mediated signaling by fungus is sufficient to activate FAK/PAX in HCECs. We proved that FAK phosphorylation correlated with the activation of its downstream PAX signaling pathway. Our data also indicates that the fungus-induced phosphorylation of FAK correlated with the physical association of β1 integrin with subsequent activation of the PAX signaling pathway. When FAK tyrosine phosphorylation was blocked with genistein, adherence was lower in the blockade group than in the non-blockade group. Recent reports demonstrate the involvement of PAX with FAK signaling pathway activation, cell migration, and signal spreading [26,27]. The interaction of FAK and PAX produces a molecular switch resulting in tyrosine phosphorylation of the remaining PAX and determines the fate of downstream signaling events [28]. So, we suspect that *F. solani* infection is associated with rapid and transient phosphorylation of PAX, followed by phosphorylation of FAK. *Fusarium solani*-induced activation of FAK is an early event and possibly a prerequisite for complete activation of FAK.

Previous data have shown that the interaction of fungus with epithelial cells results in actin rearrangement in the host cells, membrane ruffling, and cellular motility, the effects of which are both dose and time dependent [29]. We studied the effects of the FAK/PAX and FAK inhibitor, genistein, on actin rearrangement in our model system and attempted to explore the putative action mechanism of fungus. Our observations indicate that the interaction of *F. solani* with HCECs causes actin rearrangement in HCECs. The activity of the *F. solani* in altering actin arrangement was decreased by the supplementation of FAK inhibitor and the interaction of spores with the cells was reduced. This may possibly be explained through the perturbation caused by genistein to the actin polymerization.

Our results indicate that once *F. solani* spores attach to the ECM, integrin stimulation of FAK/PAX promotes adhesion and enters the cells by a process of triggered ruffling and internalization. Although the results obtained in this study showed the consequence of the interaction between mammalian cells with *F. solani*, the mechanisms of this process and its implications in the infection process still require further investigation. Further elucidation of the molecular interactions that trigger the uptake of host cells will be important in understanding this mode of pathogenesis.

## 
